# Elevated levels of cortisol in hair precede acute myocardial infarction

**DOI:** 10.1038/s41598-020-80559-9

**Published:** 2020-12-31

**Authors:** Tomas Faresjö, Susanna Strömberg, Mike Jones, Andreas Stomby, Jan-Erik Karlsson, Carl Johan Östgren, Åshild Faresjö, Elvar Theodorsson

**Affiliations:** 1 Department of Health, Medicine and Care, General Practice, Linköping University, Linköping, Sweden; 2 Psychology Department, Macquarie University, Sydney, NSW Australia; 3 Department of Health, Medicine and Care, Linköping University, Linköping, Sweden; 4 Department of Cardiology, Linköping University, Linköping, Sweden; 5 Department of Health, Medicine and Care, Public Health, Linköping University, Linköping, Sweden; 6 Department of Biomedical and Clinical Science, Clinical Chemistry, Linköping University, Linköping, Sweden

**Keywords:** Biomarkers, Cardiology, Risk factors

## Abstract

Long term stress exposure is typical for modern societies and might trigger different diseases. This case–control study reveals that persons who had suffered an acute myocardial infarction (AMI) had elevated cortisol concentrations in the month before the acute event. Middle-aged patients admitted to cardiology clinics with acute myocardial infarction (AMI) (n = 174) were compared to 3156 controls from a population-based cohort in southeast Sweden. The median Hair Cortisol Concentrations (HCC) for those who had suffered an AMI was 53.2 pg/mg compared to 22.2 pg/mg for the control group (p < 0.001). In bivariate analysis, higher levels of HCC were strongly (OR = 5.69) and statistically significantly associated with current AMI status. The discrimination of cases with AMI from controls remained statistically significant (OR = 5.04) even after controlling for established cardiovascular risk factors in a multivariate analysis. Middle-aged persons with acute myocardial infarction had significantly elevated cortisol levels during the month before the cardiac event. This was evident for both men and women. The biomarker cortisol concentration was independently and statistically significantly related to AMI. Chronic stress seems to be a new promising risk factor for AMI.

## Introduction

Acute emotional and physical stress are established risk factors for triggering myocardial infarction^1,2^. It is, however, unknown whether chronic elevated stress over periods of months is responsible for the observed increase in the risk of myocardial infarction. The empirical evidence that chronic stress is a risk factor for cardiac events is as yet scarce^3,4^. A major reason for this is that until recently we only have been able to measure acute stress and not chronic stress in humans. The individual’s response to stress includes activation of the hypothalamic–pituitary–adrenal (HPA) axis and the autonomic nervous system, including the release of the stress hormone cortisol. The prevailing plasma concentrations of cortisol are incorporated in hair as it grows from the hair root at a speed of approximately 1 cm/month, reflecting the retrospective timeline of the HPA-axis activity^5,6^. Hair cortisol concentration (HCC) is a promising biomarker of stress in populations and a potential biomarker for clinical applications in individuals^7–9^. The cumulative effects of chronic stressful situations in daily life that exceed the individual’s ability to cope cause allostatic overload^10^. Accordingly, elevated cortisol levels have been found to be associated with obesity, diabetes type II, cardiovascular risk factors, and cardiovascular diseases^3,4,9,11–14^.

We hypothesized that chronic stress, measured by HCC, is associated with increased risk of AMI. Accordingly, our aim was to investigate HCC as a reflection of retrospective HPA-axis activity in middle-aged men and women hospitalized for acute myocardial infarction compared to the HCC in a random sample of individuals from the general population in the same geographical area. Furthermore, in this study we explored the relative importance of chronic stress measured by HCC compared with established cardiovascular risk factors.

## Material and methods

### Subjects

In this case–control study the cases constitute of men and women up to the age of 65 years who were admitted for acute care to cardiology clinics (data collected 2016–2019) and diagnosed with acute myocardial infarction (ICD-10 I21). The AMI cases were included at three hospitals (one university hospital and two regional hospitals) in the region of south-eastern Sweden. Inclusion criteria were: ST segment Elevation Myocardial Infarction (STEMI) or non-ST segment Elevation Myocardial Infarction (NSTEMI). Exclusion criteria were non-Swedish speaking, and insufficient hair length (under 1 cm) on the vertex area of the scalp. The patients were invited to participate in the study at the time of discharge from the cardiology clinic. The time span from reported onset of AMI symptoms to hair sample being taken and inclusion in the study was generally 2–3 days (mean = 2.55 days, SD = 2.3). The inclusion of cases was made over a 3-year period to match the data collection of the controls. All subjects provided written informed consent and the total number of cases included were N = 174 (127 men and 47 women) with a mean age of 58 years (SD = 6).

### Control from the Swedish CArdioPulmonary bioImage Study (SCAPIS)

SCAPIS is a National collaborative project between six Swedish universities, including 30,000 randomly sampled individuals from the general population aged 50 to 65 years^15^. The SCAPIS cohort from the Linköping site for whom an additional sampling of hair was made constitute the control group in this study (data collected 2015–2018). The data collection from the SCAPIS Linköping included N = 5057 participants, of these n = 3156 participated in the HCC data collection (n = 1247 men and n = 2009 women) with a mean age of 57 years (SD = 4). Exclusion from the HCC collection among the SCAPIS controls were mainly due to insufficient hair length. Neither educational level nor the occurrence of cardiovascular risk factors differed between the HCC excluded and those providing hair samples among the SCAPIS controls. However, among those excluded (men), hypertension diagnosis was slightly more prevalent 25.2% vs. 20.4% (p = 0.006). There was no difference (p = 0.656) in recruitment rates across seasons between AMI-cases and SCAPIS controls. A lower inclusion rate was evident in the summer for both cases and controls.

### Measuring cardiometabolic risk factors and social data

All AMI cases and controls were asked to fill in a questionnaire regarding cardiovascular risk factors and social data. Further, they were asked if they had ever been diagnosed for and/or prescribed medication for Hypertension, Hyperlipidaemia, or Diabetes Mellitus. The questionnaire also included questions about previous cardiovascular and cerebrovascular diseases: Angina Pectoris, Heart failure, Coronary by-pass surgery (CABG), Percutaneous coronary intervention (PCI) or Stroke/ Transient ischemic attack (TIA). They were also asked if they had previously been hospitalized and diagnosed for myocardial infarction, and if they have a family history of myocardial infarction (either from father, mother or siblings). Autoimmune diseases like Rheumatoid Arthritis (RA), Inflammatory Bowel Disease (IBD), Systemic Lupus Erythematosus (SLE), Psoriasis and Bechterew's disease were also registered. Responses to these questions were crosschecked with medical records from the Swedish National Register of Myocardial Infarction (The Swedeheart Register). Anthropometric measurements and BMI (dichotomised under or over 30) were recorded by research nurses. Social data, such as educational level were graded on a three-point scale and social support, was defined by having someone to confide in and an indicator of socioeconomic stress was the person perceiving strained finances during the preceding year.

### Hair sampling and HCC analysis

Hair was cut from the vertex posterior of the scalp close to the skin by trained staff, in accordance with the recommendations of the Society of Hair Testing (SoHT)^16^, and because this area has a consistent growth rate^17–19^. Each hair sample had a minimum length of 1.0 cm, representing exposure to stress around 4–6 weeks in the past. The method applied for extraction and analysis of cortisol levels in hair was a competitive RadioImmunoAssay (RIA), that enable us to analyse small samples of as small as 1 cm in length^20,21^. The hair samples were then cut into smaller pieces, frozen for 2 min in liquid nitrogen, and minced together with a steel ball using a Retch Tissue Lyser II for 2 min. Methanol (1 ml) was added to each tube and the samples were extracted overnight on a moving board. Then 0.8 ml of the methanol supernatant was pipetted off and lyophilized using a Savant Speed Vac Plus SC210A, and the samples were dissolved in radioimmunoassay buffer and analysed^22^. The primary antibody used was specifically suitable for RIA (Rabbit Cortisol 3 Polyclonal Antibody, MyBiosource, San Diego, USA). The secondary antibody Sac-Cel was anti-rabbit (Sac-Cel AA-Sac1, ImmunoDiagnostic Systems Ltd, Boldon, England). Hair samples of 5 mg or more were needed in order to maintain a total inter-assay coefficient of variation below 8% for hair extraction and measurement of cortisol by the radioimmunoassay. The intra-assay coefficient of variation for the radioimmunoassay itself was 7% at 10 nmol/L.

For a random, but limited sample of n = 22 individuals, we made a methodological comparison of our RIA-method and a mass spectrometry method (LCMS/MS). This analysis showed a very high conformity between the two methods in a linear regression (Y = 0.4111 * X − 3.134 and R-square = 0.8705 p < 0.001). Furthermore, the gamma radiation used for detection in the RIA-method is not influenced by colored hair which is extracted to the methanol together with the cortisol.

The research protocol and all methods in the study were carried out in accordance with relevant guidelines and regulations. The use of human tissues and experiments in the Stressheart study was approved by the Regional Ethical Review Board in Linköping, Sweden (Dnr 2016-79-31, Dnr 2016-453-32, Dnr 2017-106-32). The SCAPIS study was approved by the Umeå Ethical Review Board (Dnr 2010-228-31M). All participants gave their written informed consent to participate.

### Statistical analysis

Quantitative measures have been described using mean and standard deviation or median and interquartile range (IQR) in the distribution of HCC. The HCC was log-transformed in some descriptive analysis, in other analysis HCC was divided into quintiles. The extent to which hair cortisol discriminates AMI cases from controls has been evaluated using unconditional logistic regression. The influence of individual risk factors, including HCC, is reported as odds ratios with a 95% confidence interval. Bivariate association with risk for myocardial infarction is presented in Table 2 and the factors identified as statistically significant risk factors were included in the multiple logistic regression model also in Table 2.

## Results

### Study participants

A general description of the included AMI cases and the SCAPIS controls in the study is shown in Table 1.

**Table 1:** Description of the AMI-cases (N = 174) and the SCAPIS-controls (N = 3 055).

	AMI cases N = 174		SCAPIS controls N = 3055		
	n	%	n	%	p-value
**Gender**					
Males	127	73.0	1147	36.3	< 0.001
Females	47	27.0	2009	63.7	
**Age group**					
< 54 years	47	27.0	1159	36.7	< 0.001
55–59 years	47	27.0	998	31.6	
60–66 years	80	46.0	999	31.7	
Previous myocardial infarction	36	20.7	40	1.3	< 0.001
Heredity for myocardial infarction	49	36.0	211	6.9	< 0.001
Previous cardiovascular or cerebrovascular disease	45	25.9	116	3.8	< 0.001
Diabetes type 2	31	17.8	140	4.6	< 0.001
Hypertension diagnosed/treated	69	40.1	593	19.4	< 0.001
Hyperlipidaemia diagnosed/treated	39	22.7	288	9.4	< 0.001
Obesity BMI > 30	40	23.0	643	20.4	0.413
Present smoker	23	13.2	289	10.0	0.179
Autoimmune diseases	7	5.1	171	5.6	0.837
Low education	39	22.4	263	8.6	< 0.001
Financial strain last year	14	10.3	102	3.3	< 0.001
Lack of social support	13	7.6	169	5.6	0.270

The mean age of the male AMI cases was significantly higher p < 0.001 (mean: 58.06 SD = 6.1) than the male SCAPIS controls (mean: 56.06 SD = 4.5). For women, there was no difference (p = 0.857) in age between the AMI cases (mean 56.85 SD = 6.2) and the SCAPIS controls (mean 56.73, SD = 4.5). As shown in Table 1, most of the traditional cardiovascular risk factors measured were substantially more prevalent among the AMI cases than the SCAPIS controls. However, no difference was found in the occurrence of angina pectoris among the AMI cases (4.3%) and the SCAPIS controls (3.8%), p = 0.674. There were no differences in the prevalence of obesity, in smoking habits or autoimmune diseases between the AMI cases and the SCAPIS controls in this study. Low education and strained financial difficulty in the preceding year were significantly more prevalent among the AMI cases.

### HCC for those previously affected by myocardial infarction

AMI cases who had previously experienced a myocardial infarction (n = 36) had a slightly elevated median HCC of 62.3 pg/mg (IQR 33.4–177.1), compared to those with a first-time AMI (n = 138) median of 51.5 pg/mg (IQR 23.3–127.5) (n = 138), but this difference did not reach statistical significance (p = 0.801). SCAPIS controls with a previous AMI (n = 40) also had a slightly elevated HCC median 29.8 pg/mg (18.5–112.6), compared with those with no AMI experience (HCC median 22.3 pg/mg (IQR 14.8–44.2) (n = 3055). This difference did not reach statistical significance (p = 0.113).

### HCC for myocardial infarction patients and controls

**Figure 1:**
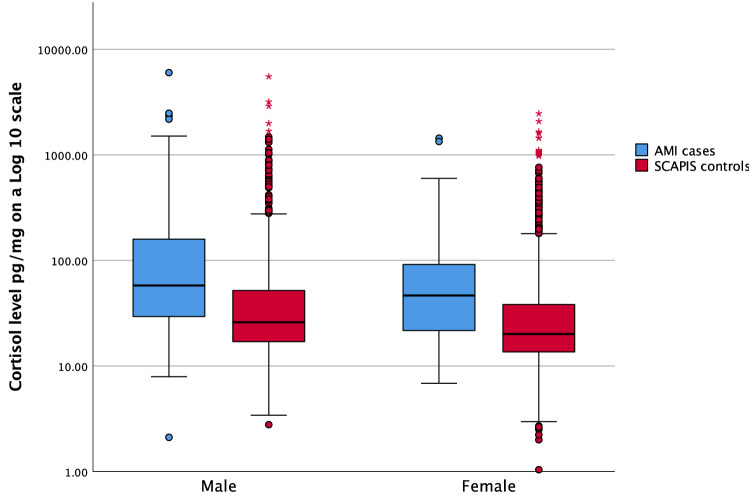
Hair cortisol levels for AMI-cases and SCAPIS-controls.

The median HCC for those who had suffered an AMI was 53.2 pg/mg (IQR 26.4–136.2) compared to 22.2 (IQR 14.8–43.6) pg/mg for the control group (p < 0.001). For men who had suffered an AMI the median was 57.8 pg/mg (IQR 29.0–167.6) compared to 25.9 pg/mg (IQR 17.0–52.0) for control group males (Fig. 1, p < 0.001). Correspondingly the HCC for women who had suffered an AMI was 46.6 pg/mg (IQR 20.5–100.8) and in the control group women 20.1 pg/mg (IQR 13.6–38.2) (p < 0.001), see Fig. 1. Hair cortisol (log_10_) provided statistically significant discrimination between AMI cases and SCAPIS controls in men (OR 1.70, 95% confidence interval 1.47, 1.95, p < 0.001) and in women (OR 1.70, 95% confidence interval 1.37, 2.12, p < 0.001).

**Figure 2:**
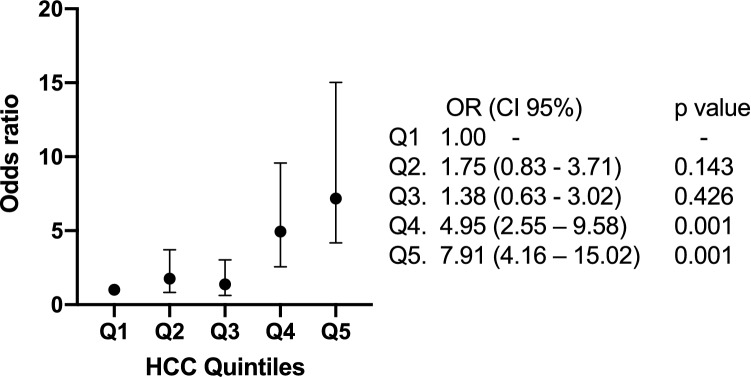
Odds ratios for AMI in quintiles of hair cortisol concentrations.

The cortisol levels divided into quintiles and the OR for AMI are illustrated in Fig. 2. With this subdivision of the HCC it becomes clear that the relationship between HCC and AMI is not linear, there is minimal increase in risk of AMI across quintiles 1–3 but the probability for AMI increase greatly in quintiles 4 and 5 of HCC.

We made an additional analysis were AMI cases using glucocorticoids (n = 33) were removed. A new analysis of the remaining n = 141 AMI cases compared to the controls (n = 3156) revealed that the median cortisol levels for the AMI cases not using glucocorticoids was only slightly lowered (53.19 pg/mg compared to 47.05 pg/mg).

### Biological stress in comparison to traditional cardiovascular risk factors

Bivariate analysis of potential cardiovascular risk factors is shown in Table 2. The strongest bivariate risk factor was previous MI followed by previous cardiovascular or cerebrovascular diseases and heredity. The two highest quintiles HCC showed a high OR = 5.69 for AMI. Obesity, autoimmune disease, present smoker, and lack of social support were not included in the bivariate analysis as there were no significant difference in these factors between the cases and controls. The factors that were statistically significant in the bivariate analysis where further included in the multiple logistic regression, also shown in Table 2.

**Table 2:** Bivariate and multivariate logistic regression of factors associated to risk for myocardial infarction, data for AMI-cases (N = 174) and SCAPIS-controls (N = 3 055).

Risk factors	Bivariate analysis		Multivariate logistic analysis	
	Odds ratio (CI 95%)	p-value	Odds ratio (CI 95%)	p-value
High cortisol levels (Q4 + Q5 vs. Q1 + Q2 + Q3)	5.69 (3.54–9.15)	< 0.001	5.04 (3.12–8.16)	< 0.001
Male gender	4.73 (3.36–6.67)	< 0.001	4.26 (4.26–2.70)	< 0.001
Age > 60 years	1.84 (1.35–2.50)	< 0.001	N/A	
Previous myocardial infarction	19.66 (12.15–31.83)	< 0.001	9.21 (4.66–18.17)	< 0.001
Heredity for myocardial infarction	7.56 (5.18–11.02)	< 0.001	8.12 (5.10–12.93)	< 0.001
Previous cardiovascular and/or cerebrovascular disease	8.87 (8.87–6.03)	< 0.001	6.11 (3.43–10.88)	< 0.001
Hypertension diagnosed/treated	2.77 (2.02–3.82)	< 0.001	1.90 (1.23–2.95)	0.008
Hyperlipidaemia diagnosed/treated	2.82 (1.93–4.11)	< 0.001	N/A	
Diabetes type 2	4.53 (2.97–6.92)	< 0.001	2.47 (1.28–4.79)	0.006
Low education	3.09 (2.10–4.50)	< 0.001	2.44 (1.42–4.18)	0.002
Financial strain last year	3.34 (1.85–6.00)	< 0.001	3.62 (1.67–7.84)	0.001

Logistic Regression model: Exp (B) = 21.50, Wald = 1223.3, p < 0.001.

Non-significant factors excluded in the reduced multivariate model: Smoking, BMI-Obesity, Autoimmune disease (Inflammatory bowel disease, RA, Bechterews, SLE, Psoriasis), Age over 60 and Hyperlipidaemia.

The discrimination of cases with AMI from controls by HCC remained statistically significant after controlling for other potential cardiovascular risk factors. Previous MI, prevalent cardiovascular or cerebrovascular disease together with a family history of AMI yielded the strongest associations with being a current AMI case rather than a control (Table 2). Diagnosed diabetes mellitus type 2 and hypertension together with social factors such as low educational level and financial strain were also independently associated with AMI. Higher levels of HCC were strongly and independently associated with current AMI status with an OR = 5.04 (Table 2).

## Discussion

In this study we found that middle aged men and women who had suffered an AMI had significantly elevated biological stress during the month before the infarction, compared with controls from the general population. Chronic stress measured by HCC was independently and statistically significantly associated with AMI, even when established cardiovascular risk factors were controlled for. These findings indicate that chronic biological stress, as measured by HCC, may be a new and clinically significant risk factor for AMI.

Working life generates stress, one of the main factors that increases coronary heart disease risks in our society today^23^. In middle age, the general risk of myocardial infarction is lower for women than for men, and this may have led previous studies in this field to only include men^3,4^. Our study revealed that chronic stress seems to have an impact on AMI and cardiovascular risks for both genders. The possible effect of chronic stress measured by HCC on AMI risk has been reported previously in two small studies of males in selected samples^3,4^. However, these studies concluded that population-based larger epidemiological studies also including females was warranted, to establish this association.

Some of the traditional cardiovascular risk factors such as hypertension and hyperlipidaemia were associated with relatively small effect sizes in our multivariate model, compared to other factors such as previous MI or heredity for MI. Hyperlipidaemia did not even reach statistical significance. However, these factors must still be considered as important risk factors in the etiological causal chain for AMI. These factors are also linked to chronic stress and reducing upstream risk factors may be effective in reducing risk. Participants with a history of high blood pressure and high lipids might previously have received medical treatment for these conditions, so therefore their AMI risks might have been reduced. The low impact of smoking on AMI risk in this study might reflect a widespread smoking cessation for both men and women over the last decade in Sweden. Also, BMI as a traditional risk factor did not appear to have any significant impact on AMI risk in this study. As expected, male gender presented a high OR for AMI in this middle aged population^1^.

The increased HCC before an AMI is supported by both psychosocial and biological mechanisms^14^. Supraphysiological cortisol levels caused by Cushing’s syndrome or glucocorticoid treatment are linked to increased prevalence of both cardiovascular risk factors and myocardial infarctions^24–26^. Furthermore, slightly elevated cortisol levels are also associated with cardiovascular risk factors^3,14^. Thus, it is plausible that increased cortisol levels cause metabolic derangements, which leads to atherogenesis and, in the long term, development of myocardial infarctions. However, increased cortisol levels also have direct effects on the cardiovascular system, effects including increased vascular contractility, inhibited angiogenesis, and increased platelet activation leading to thrombosis^25^. Notably, these direct cardiovascular effects were present among patients who reported that an emotional event had preceded their myocardial infarction in particular^27^. The analysis of the present data was performed on group level. The data indicates but does not establish that HCC as marker for chronic stress is a risk factor for cardiac infarction at an individual level.

Psychological distress can be associated with increased cardiovascular risk^2,28,29^. It would be interesting to further elaborate the relationship between perceived stress, HCC and risk for AMI. Our study design is not ideally suited for investigating perceived health and psychosocial factors due to the risk of recall bias among the AMI-cases, which might not be as evident in the control group. The cases were inpatients on a cardiology ward who just recently have had AMI which can be potentially life-threatening. This, in itself, can cause significant stress and might effect their mental state and how they recall the period prior to the infarction. The control group are in a very different situation, they are randomly selected from the general population. The emphasis in this study is on the biological stress marker in comparison to established CVD risk factors and we have therefore not focused on factors that reflect perceived stress and health. The HCC reflects the period before the infarction and is not affected by the bias that the situation around the infarction causes.

The HCC was analysed with an established RIA method. The primary reason for using this method is that it has a very low limit of detection. This enables the measurement of cortisol in 5 mg samples of hair that can be taken with no inconvenience to the participants.

When measuring biomarkers such as HCC there might be a fraction of the results characterized as outliers in the distribution when these exceeded the mean + 3 SD. The HCC distribution was divided into quintiles in the statistical analysis and this minimizes the potential undue influence of outlying values. A possible risk that might have occurred is the interaction of the use of glucocorticoids ointments with the RIA antibody, which could falsely increase the measured cortisol levels. For the participants in the study we have information of usage of glucocorticoids in the cases, but unfortunately not in the control group. As described in the result section, the original (unadjusted) difference in cortisol levels between cases and controls prevail even after those with glucocorticoid usage were removed. This supports the notion that glucocorticoid usage is not a driver that could explain HCC and myocardial infarction risk.

The controls in this study are representative of the general population, and therefore include a proportion of individuals with prevalent cardiovascular risk factors and previous myocardial infarction. These individuals were not omitted from the analyses, since we also wanted to shed light on the relative importance of earlier cardiovascular risks in relation to HCC, and such individuals are a normal part of the general population. Cushing’s disease was not an exclusion criteria as it is very rare^30^ but it is unlikely that any single case of Cushing’s would have any effect on the results or conclusions in the study. Seasonal variation in HCC levels might affect the results, but the sample collection dates for both the AMI cases and the SCAPIS controls were evenly distributed during the same 3-year period in the same geographical region in Sweden.

A general limitation in studies applying the HCC method is that people with insufficient hair length are excluded from the analysis. This limitation could have some impact on the results if baldness is related to chronic stress, but no evidence is available to this theoretical confounder. An analysis of the non-attendees in the SCAPIS cohort shows that they did not differ from the participants concerning educational background or cardiovascular risks.

Prodromal symptoms including chest pain, shortness of breath, fatigue, sleep deprivation, is a part of the natural cause of myocardial infarction and we cannot rule out that for a time preceding the AMI, such symptoms might have occurred. However, studies have shown that most patients do not attribute their prodromal symptoms to impending MI^31^. It is possible that prodromal symptoms might increase the HCC levels by biological mechanisms, but such possible mechanisms are not fully elaborated, and further research is warranted. Prodromal symptoms might also affect the individual’s perception of stress and thereby raise the HCC levels. Many earlier studies show that there are no consistent associations between perceived stress and increased HCC levels^13^. The month preceding the cardiac event some individuals with prodromal symptoms might have seek either primary health care or hospital care. If angina was presented at such health care visits these diagnoses have been registered and considered in our multivariate analysis. The occurrence of angina pectoris was low in both AMI and SCAPIS controls and there was no difference between the groups. A major Canadian study found that only a small proportion of patients (15.9%) with Acute Coronary Syndromes seek medical attention for prodromal symptoms in the 90 days before the cardiac event^32^. We have not analysed data of hospitalization or primary health care visits the month preceding the AMI, which is a limitation in this study. It is though important to keep in mind that the study population is middle age and hospitalisation in this age group in less common than in older and more fragile populations. The male AMI cases were slightly older (mean age 58 years) compared to the SCAPIS controls (mean age 56 years), no age difference for females. These minor age difference do not necessarily lead to more co-morbidity or extended hospitalization.

A major strength in this study design is that we were able to exactly date the acute cardiac event, and our hair sample is taken on average 2.5 days after this event. The last 5 to 10 days of hair growth lies just beneath and under the scalp, so the increased stress as a possible direct effect of the myocardial infarction itself could therefore not interfere with the hair sample in our analysis^20^. The fatal cases of AMI were not included in our sample, which could be a limitation. In Sweden, the rates of fatal AMI cases is around 8 to 12% yearly of all AMI cases in the ages below 65 years^33^. Another major strength is the large SCAPIS control group from the general population examined with a standardized and detailed protocol. However, the controls may confer a risk of selection bias since we know from the SCAPIS pilot study that low socio-economic status was associated with lower participation rate^34^.

## Conclusions

Biologically measured chronic stress was significantly elevated during the month before an acute myocardial infarction for both middle-aged men and women, compared to controls in the general population. The biomarker HCC was independently associated with AMI, even after adjustments for the major established cardiovascular risk factors. This study shows that chronic stress seems to be a new and significant risk factor for AMI.
